# Development and adaptation of a strength-based job interview training tool for transition age youth on the autism spectrum using community engaged methods

**DOI:** 10.3389/fpsyt.2023.1098334

**Published:** 2023-09-14

**Authors:** Helen M. Genova, Mikayla Haas, Yu-Lun Chen, Heba E. Elsayed, Robert E. McGrath, Matthew J. Smith

**Affiliations:** ^1^Kessler Foundation, East Hanover, NJ, United States; ^2^Rutgers New Jersey Medical School, Newark, NJ, United States; ^3^School of Psychology and Counseling, Farleigh Dickinson University, Teaneck, NJ, United States; ^4^School of Social Work, University of Michigan, Ann Arbor, MI, United States

**Keywords:** autism, job interview, positive psychology, intervention, mixed methods, strength-based, web-based

## Abstract

**Introduction:**

Kessler Foundation Strength Identification and Expression (KF-STRIDE) is a strength-based job interview training tool developed for young adults on the autism spectrum. The intervention is based on a positive psychology framework to increase knowledge of character strengths, and how to relate them to a future employer. The current study sought to evaluate the acceptability, usability and feasibility of KF-STRIDE, as well as to guide adaptations to improve the tool’s ability to meet the needs of those on the spectrum.

**Methods:**

Mixed methods (post-intervention surveys, and semi-structured interviews with key stakeholders) were used to inform the evaluation and consequent adaptations of KF-STRIDE.

**Results:**

The major findings of the study were that KF-STRIDE was found to be largely acceptable and usable. Importantly, however, our qualitative analysis revealed modifications that could help to better suit the needs of young adults on the spectrum, which included the incorporation of additional skills (i.e. etiquette, practicing hygiene) and more opportunities to practice job interviewing. Thus, we altered the implementation of the intervention to be web-based to improve accessibility. We incorporated the presence of an animated character to deliver the content, to eliminate the need for a highly trained interventionist.

**Discussion:**

KF-STRIDE was modified to increase access by incorporating feedback from the autism community. Future directions include assessing the efficacy of KF-STRIDE in young adults on the spectrum to identify whether employment outcomes are improved after using the tool.

## 1. Introduction

Approximately 80% of persons on the autism spectrum are unemployed, and the highest unemployment risk tends to affect youth within 2 years of high school graduation (1–3). One obstacle toward gaining employment is the job interview, a complex conversation where one needs to effectively convey their interest in a job as well as their personal strengths to do well in the job (4). Meanwhile, transition-age youth on the spectrum experience significant social communication challenges and social anxiety that may be a barrier to successfully and efficiently conveying this important information while establishing rapport with a future employer (5–7). Notably, more than 90% of employed young adults on the spectrum engaged in special education pre-employment transition services (Pre-ETS) completed a job interview prior to getting their job (8). Moreover, job interview training was identified as a critical component of job readiness training that is federally mandated for inclusion in special education Pre-ETS (9). However, Pre-ETS and adult vocational rehabilitation services currently lack access to an evidence-based job interview training intervention (10).

Behavioral interventions for individuals on the autism spectrum, including those for obtaining employment, have focused largely on fixing what individuals are doing wrong (attempting to ameliorate “impaired” functioning) (11–13). While these treatments may be necessary for certain positive outcomes, it has been argued that this approach may not help an individual to become aware of how to use their strengths (13–19). For example, in our research we have observed that job interview skills can be improved by repeated role pray practice, but without lessons on one’s individual strengths, a person can continue to have difficulty expressing their strengths to a future employer (20). A strength-based intervention attempts to harness the strengths of an individual on the spectrum in treatment (13, 14, 19, 21). While “strengths-based” can be defined a number of ways, it is generally thought to consider one’s strengths, skills or talents in its approach.

The current paper describes the development, evaluation and modification of a novel job interview intervention, Kessler Foundation Strength Identification and Expression (KF-STRIDE^®^), which was developed using a positive psychology-based framework to focus on strengths. The field of positive psychology focuses on the cultivation of what a person *can do*, as opposed to fixing what is deficient in a person (i.e., what a person cannot do) (22). One of the most well-established tenets of positive psychology is the study of character strengths. Much of this work has focused on 24 desirable traits that every individual expresses to differing degrees (23, 24). The two main goals of KF-STRIDE are to help young adults on the spectrum: (1) *identify* their character strengths, and (2) *express* them to others in a socially appropriate manner.

In KF-STRIDE, users identify their personal character strengths based on the *Aware-Explore-Apply* character strength interventions framework, consistent with published principles of positive psychology (25, 26). All participants take a validated assessment of character strengths, the *VIA* Inventory of Strengths (27) (*VIA*-IS) to identify personal character strengths (*Aware*). Participants identify when they have used those strengths before (*Explore*), and describe scenarios in which they may use their strength in the future (e.g., during employment) (*Apply*).

Improving character strength identification (by teaching individuals to learn and cultivate character strengths) has been used across clinical populations [such as individuals with visual impairment (28), brain injury (29), and depression (30)] and in the general population (31). Mounting evidence indicates that character strength identification has significant clinical benefit, including improved mood (32) and life satisfaction and well-being (33), and it has been used in employment settings in the general population (34). Despite clinical benefits of character strength identification, few studies have applied this positive psychology approach to autism (12, 35). Thus, KF-STRIDE takes a unique approach by focusing on character strengths in young adults on the spectrum within the context of job interview training.

The purpose of the current user experience study was to evaluate and further develop KF-STRIDE using mixed methods. Our goal was to evaluate autism community stakeholder perceptions of expected implementation feasibility, usability of KF-STRIDE, and post-implementation acceptability. The feedback from stakeholders was used to modify KF-STRIDE into a new tool which better suits the needs of the autism community.

## 2. Methods

### 2.1. Approach

**Figure 1:**
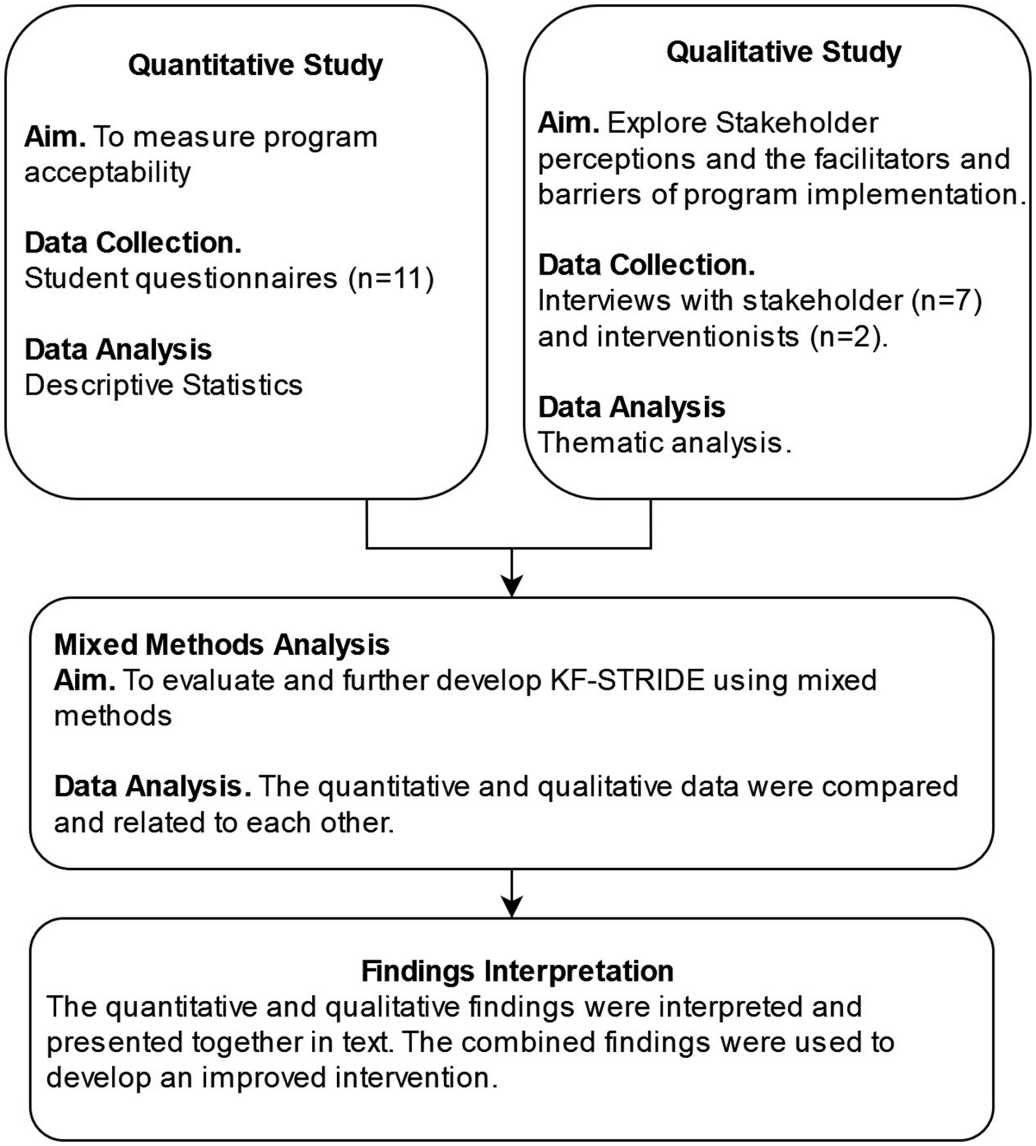
Study design and interpretation.

Using a parallel, mixed methods data collection approach (36), our evaluation included collection of both quantitative and qualitative data. We analyzed the qualitative data to support interpretation of quantitative data regarding the acceptability of KF-STRIDE. Specifically, our qualitative data explored broader indicators of program acceptability, including stakeholders perceptions towards the intervention acceptability, usability, and feasibility. Using a convergent design (37), data were integrated during interpretation (Figure 1).

### 2.2. Quantitative data to evaluate user experience: program acceptability

#### 2.2.1. Participants

The quantitative study included 11 young adults on the spectrum. Autism diagnosis was confirmed through either medical or educational record review. Participants were recruited using social media, local support groups, partnerships with special needs schools, and Research Match.^1^ Most respondents were male (*n* = 8, 72%). The age of respondents ranged from 17 to 24, with a mean age of 20.64, SD = 2.54. Regarding race, the participants were predominantly White (63.6%), while 18.2% were Black/African American and 18.2% Asian. As for educational level, 9.1% had 11 years of education, 54.5% had 12 years, 9.1% had 13 years and 18.2% had 14 years education and 9.1% had 15 years education, see Table 1.

**Table 1:** Demographics for participants.

Variable	Categories	*n* (%)
Race (*n* = 11)		
	White	7 (63.6%)
	Black/African American	2(18.2%)
	Asian	2 (18.2%)
Education (*n* = 11)	(Grade in Years)	
	11	1 (9.1%)
	12	6 (54.5%)
	13	1 (9.1%)
	14	2 (18.2%)
	15	1 (9.1%)

The acceptability data analyzed for this article were collected as part of a Randomized Controlled Trial (RCT) evaluating KF-STRIDE among young adults on the spectrum. KF-STRIDE effectiveness data from this RCT are still being collected and will be published separately. Twenty-two participants participated in the RCT, of which 11 were randomized to the experimental group who received KF-STRIDE in combination with Virtual Reality Job Interview Training (VR-JIT); a computerized job interview simulator with established efficacy and effectiveness among young adults on the spectrum (20, 38, 39). Participants received 9 h of VR-JIT, followed by 3 h-long sessions of KF-STRIDE given on separate days. Only the experimental group’s quantitative data were included in the current analysis, as only they received the intervention. The quantitative data collection was reviewed and approved by Kessler Foundation’s Institutional Review Board. All participants provided informed consent.

#### 2.2.2. Measures

In order to measure participant acceptability of KF-STRIDE, participants were asked on a scale of 1–5 (1 = “not true at all,” 2 = “a little true,” 3 = “somewhat true,” 4 = “true,” 5 = “very true”) about their opinion of KF-STRIDE and whether it was (1) easy to use, (2) enjoyable, (3) helpful in preparing for a job interview, (4) improved their confidence in job interviews, (5) helpful in learning new things about themselves, and (6) helpful in making them feel good about themselves. This questionnaire was given to participants at a follow-up testing session within 2 weeks of their KF-STRIDE completion.

#### 2.2.3. Description of intervention (KF-STRIDE)

The KF-STRIDE Intervention was designed to be implemented over three individual sessions, each is 1 h long that were administered on 3 different days. The content was developed by the PI (HMG) using the character strength identification intervention *Aware-Explore-Apply* model from the field of positive psychology (25, 26). Sessions were attended by a student and a trained interventionist who administered the intervention. Each session was an hour long, and delivered on different days (with ideally at least 2 days between sessions) to allow for homework completion. Each step of the *Aware-Explore-Apply* model was delivered by the interventionist, who read scripted didactic content while displaying visual stimuli *via* PowerPoint.

Session 1 (*Aware*) provided the participants with an introduction to 24 character strengths identified by the field of positive psychology. Participants completed the *VIA*-IS, a self-report questionnaire intended to measure an individual’s possession of character strengths. The *VIA*-IS is freely available online and its psychometric properties have been well-established (40). Then trainees received a list of their 24 character strengths in rank order with the top 3 representing “signature strengths” (i.e., their strengths used most often). Participants answered self-reflective questions related to the signature strengths to encourage understanding of how they use their strengths in their daily lives. Participants were then given a homework assignment called “strengths-spotting,” in which the participant kept a brief daily journal describing how they used their signature strengths that day. “Strengths-spotting” is a common intervention tool in character strength interventions to complement the *Aware-Explore-Apply* model, as it helps increase the participant’s awareness of how they possess and utilize their strengths daily.

Session 2 (*Explore*) taught the participant to identify how they can use their strengths in different situations. Participants were provided with three different scenarios and asked how they would use their strengths to help them handle the situation. For example, in one scenario, the participant was told: “*You walk into a cafeteria and see two friends having an argument. One of them looks very angry. How would you use one of your strengths to handle the situation?*” Based on participant responses (e.g., “I would use my strength of perception to help my friends understand that they have different points of view,” or “I would use my strength of judgment by staying uninvolved and letting them take care of it themselves.”), the interventionist provided supportive feedback, which included asking for clarification if the strength usage examples were unclear or lacking detail.

Participants were then asked to apply their strengths to real job scenarios. This lesson taught participants that *any* character strength can be applied to *any* job. To demonstrate this concept, they first picked a preferred job and then identified different tasks completed by a person in that job (both pleasant and unpleasant). Then, participants were asked to apply their strengths to each of those situations (“I would use my strength of honesty when working with money,” or “I would use my strength of teamwork by helping my co-workers.”). This allowed the participants to explore and identify how they could use their strengths in a potential job scenario.

Session 3 (*Apply*) focused on practicing how to answer job interview questions by talking about strengths. Participants were taught a three-step method on how to form a successful interview answer, which consists of: (1) Envisioning their top three strengths, (2) Envisioning how those strengths are important for the job they are applying for, and (3) Formulating a successful job interview answer. Specifically, participants practiced using this method with a few example occupations (e.g., cashier, a server) and then the interventionist gave them feedback (e.g., asked for clarity or to phrase it differently if a strength was not obviously applied). After participants understood how to use this method and produce a successful answer, they participated in a practice interview with a filmed actor on a pre-recorded video. The participants were asked to imagine that they were on a real interview and to answer as they would if they were really interviewing with someone. The interviewer asked questions such as “what are your biggest strengths?” and “why do you think you’d be a good fit for this job?” After each answer, participants were given immediate feedback by the interventionist related to the clarity of their answer, and whether they adequately described their strengths. To monitor fidelity, interventionists completed an adherence checklist, including notes of protocol deviations.

#### 2.2.4. Data analysis

Quantitative data were analyzed using SPSS. Responses from the treatment acceptability rating form were collapsed to create binary variables where reports of “not true at all,” and “little true” were coded as a “0″, and “true,” “very true” were coded as a “1″. Percentages of participants who endorsed scores of 1 (true, very true) are provided in the results.

### 2.3. Qualitative data: stakeholder and interventionist perceptions regarding KF-STRIDE

There were two stages of qualitative data collection. In stage 1, qualitative data were collected from key stakeholders from the autism community. In stage 2, two interventionists were interviewed. Their data were analyzed separately, as described below. The qualitative study was performed to inform the development of KF-STRIDE without intention to publish, so stakeholder consent was not obtained at the time of the semi-structured interview. When the decision was made to publish these data, the study team made every attempt to contact the stakeholders to ask permission to use their data and obtain consent. All but two could be reached and consented. Due to the importance of the data, the qualitative data of these two participants will be included from the group analysis but no direct quotes will be presented.

#### 2.3.1. Participants

In stage 1 of the qualitative data collection, seven stakeholders were interviewed, including a young adult on the spectrum, an unemployed adult on the spectrum, a parent of a young adult on the spectrum, a licensed clinical social worker specializing in autism, a clinical psychologist specializing in autism, a special education teacher primarily working with young adults on the spectrum, and a scientist who is an expert in clinical intervention development and evaluation, see Table 2 for demographics. In stage 2 of the qualitative data collection, two interventionists trained to administer KF-STRIDE were interviewed. Both were bachelor’s degree level research assistants at the Kessler Foundation.

**Table 2:** Stakeholder demographics.

Stakeholder type	Age	Gender
Youth on the spectrum	18	Male
Adult on the spectrum	31	Male
Parent of teen on the spectrum	48	Female
Licensed clinical social worker	58	Female
Clinical psychologist	52	Female
Special education teacher	55	Female
Scientist	49	Female

#### 2.3.2. Interview procedures

In stage 1, each stakeholder was interviewed by the PI with the interviews audio recorded and transcribed. Prior to the interviews, participants watched a 30-min pre-recorded presentation about KF-STRIDE regarding its development, structure, materials and implementation. The semi-structured interview questions were designed to capture stakeholder perceptions of potential barriers and potential facilitators of implementation process outcomes as defined by Proctor et al. (33) (e.g., adoption, feasibility, acceptability, etc.). Sample questions included: *“What did you like about KF-STRIDE?*,” and “*What did not you like about KF-STRIDE?.”* Further, to address feasibility, stakeholders were asked *“On a scale, of 1–10, if KF-STRIDE was commercially available and of little or no cost to you, how likely would you be to use KF-STRIDE for yourself or for the person or people in your life on the autism spectrum?”* Finally, the stakeholders’ feedback was collected concerning possible adaptations to the implementation process and the intervention *(“What other skills could be covered by KF-STRIDE if it were expanded?”; “How can KF-STRIDE be changed or modified to help autistic youth?”).* In stage 2, the PI conducted semi-structured interviews with two interventionists related to content, usability, feasibility, and barriers and facilitators to implementation.

#### 2.3.3. Data analysis

In stage 1, stakeholder interview data were transcribed and imported into NVivo 12 for coding and analysis. Using a deductive approach, the PI (HMG) and an NVivo consultant identified codes (themes) informed by our research questions. Each line of the transcribed semi-structured interview was manually read and coded with contextual content to the codes identified by the PI and NVivo consultant. Members of the study team (REDCATED) refined coding through iterative reviews and discussions.

In stage 2, during and after interviews with interventionists the PI made detailed notes on perceptions of the interventionists related to implementation, content, usability and feasibility. These notes were discussed among members of the study team (HG, MH, Y-LC, and HE) and integrated into the interpretation that guided subsequent adaptations to KF-STRIDE.

## 3. Results

### 3.1. KF-STRIDE acceptability

The quantitative data revealed that 63.6% of the young adults on the spectrum found KF-STRIDE to be enjoyable. Qualitative data revealed that KF-STRIDE’s focus on strengths and employment preparation received positive feedback from stakeholders. The quantitative and qualitative data were in agreement related to KF-STRIDE’s ability to help facilitate job interview preparation. The quantitative data found that 72.7% of young adults on the spectrum found it helpful to prepare for a job interview, while 81.8% found it helpful in improving their confidence for a job interview. The stakeholders echoed this finding. For example, the psychologist commented, “I felt that this tool would be very good in aiding [young adults] to be able to accurately represent themselves to potential employers, and to give them some insights into what their strengths are.” Stakeholders discussed the need to support young adults on the spectrum to identify and express their strengths in job interviews: e.g. “I really like [the emphasis on strengths]…I feel like it would balance out probably a lot of the programs that are already out there where we are telling kids what not to do versus put your best foot forward and being able to convey that to a potential employer.” (Teacher).

Stakeholders cited the importance of strengths for personal growth, coping, and wellbeing. The social worker commented, “if you can know that the people you are working with recognize your strengths and you can see your strengths and really build on those strengths, then it’s easier to deal with difficulties because you are coming from a strengths perspective.” The scientist echoed that more interventions should focus on strengths because “in ASD, there are very clear strengths and I think by focusing on those strengths, you’ll be able to improve someone’s overall outcome and overall quality of life.” Quantitative data supported the stakeholder’s perceptions: 63.6% of KF-STRIDE participants found it to be helpful in learning things about themselves that they did not know about and 90.9% found it helpful in making them feel good about themselves, see Table 3.

**Table 3:** Usability and acceptability of KF-STRIDE.

		*n* (%)
Usability (*n* = 11)	Easy to use	8 (72.7%)
Acceptability (*n* = 11)	Enjoyable	7 (63.6%)
	Helped to prepare for job interview	8 (72.7%)
	Helped in improving confidence for a job interview	9 (81.8%)
	Helped to learn more about oneself	7 (63.6%)
	Feel good about oneself	10 (90.9%)

The parent and teacher further stressed the need to shift to a strength perspective when supporting young adults on the spectrum into employment:

*Ultimately what led us away from ABA therapy was the focus on everything that was a deficit, I found that very depressing. I think by the time our guys are getting to the point that they're looking for jobs, they need to pay attention to what they're good at and they have a lot of interests, once shown how those interests can be tied to job skills, I think they would do a very good job with their creative minds explaining how those things could be assets.* (Parent)

*As they get into high school, you really focus on what they're really great at.…. It's really getting kids to understand what everybody needs from that position and then knowing, "Hey, what you’re really good at." You might be better suited for this. … No one wants to go to work every day, with ASD or not, with something you're not good at.* (Teacher)

The stakeholders appreciated that KF-STRIDE provided specific and practical guidance on how to identify strengths relevant to the youth and their intended careers, as opposed to existing job training programs that offer only general advice: “I think that this is much more specific than any other programs I’ve seen. I do have some kids that go into post-high school programs, and they do some kinds of job skills assessment, but I do not think anything was as in-depth as this.” (Social worker).

Stakeholders identified that KF-STRIDE is designed as a tool that can help support interview preparation among youth on the spectrum. The two individuals on the autism spectrum felt that KF-STRIDE was unique, innovative and would benefit young adults looking for a job. The parent commented that KF-STRIDE could help prepare and motivate young adults on the spectrum to interview for jobs by breaking down the expectations and emphasizing their strengths:

*This is kind of a grown-up version of a social story: …breaking down what an interview would look like, what you say, what to expect, what's expected behavior versus unexpected behavior. But the fact that you're speaking to them on an adult level and you're having them emphasize their strengths… I think they would respond very positively to and be excited about the prospect of getting to do that in real life with a real interview…* (Parent)

Other than the potential for helping job preparation, participants also noted the potential benefits of KF-STRIDE could generalize to promoting strengths identification and supporting interactions in broader social scenarios (e.g., workplace conversations):

*Once they've utilized these skills and they've seen how it works, I think it could be utilized in a way where once that is sort of internalized, that it's a process they're comfortable with, you could use that process … in relationships, for social success. So there are a number of different facets of life where I think this particular guideline or this methodology could be used in different ways.* (Psychologist)

*I think there are many situations in life, probably starting in mid-adolescence, where we're in a situation where we have to discuss ourselves with strangers and I think this would help in any of those social situations. So, you might focus the treatment on the interview process, but then a discussion could also be around where else can you apply these skills once you have them.* (Scientist)

### 3.2. KF-STRIDE usability

Of the participants in KF-STRIDE, 72.7% found it easy to use. A few stakeholders commented that KF-STRIDE’s structured and concrete curriculum was particularly helpful to foster learning among youth on the spectrum: “What I particularly liked about it was the structured element of it. Having done ABA with my son and knowing how much his needs require structure, I thought the way it was laid out was very structured, very clear, and very much in line with how someone like him learns” (Parent). Stakeholders also appreciated KF-STRIDE’s strength evaluation tool (i.e., the *VIA*-IS). The teacher noted: “What I liked about it was … [individuals] were able to take an assessment that was quick and identify some strengths.” Regarding factors that might limit usability, the interventionists described the intervention as “too short” and that the content was delivered “too quickly,” and thus more time could be devoted to additional content delivery and skills (i.e., etiquette, personal hygiene). One interventionist suggested allowing participants to change their strengths if they did not want to focus on their signature strength. They mentioned that some of the visual stimuli and content could be perceived as immature for an older population of young adults.

### 3.3. KF-STRIDE feasibility

Regarding feasibility, six out of seven stakeholders rated their likelihood to use KF-STRIDE if it was free to them and commercially available as a 10 out of 10. One stakeholder rated this item as a 7/10 and indicated that it would need to be at a convenient time and place to participate in the program due to their having to commit to such a program outside of a school environment. The interventionists indicated that KF-STRIDE administration could be difficult at times given the major role of the interventionist. Specifically, the required interventionist training, the amount of manualized materials, the powerpoint files, the scripted content, and the standardized feedback felt burdensome, and could be confusing/inconvenient/unappealing to future KF-STRIDE interventionists such as teachers and job coaches. Further, interventionists voiced concern that depending on the expertise, training and personality of the interventionist, the intervention may be delivered in inconsistent ways, which could limit the reliability of administration and ultimately the effectiveness of the intervention.

### 3.4. Suggested adaptations

Stakeholders made several suggestions for how KF-STRIDE might better meet the needs of youth on the spectrum. Specifically, they suggested the following adaptations: extending the program length; expanding the range of targeted skills; and integrating KF-STRIDE into a variety of services, such as post-high school programs (e.g., vocational or job readiness programs), commercially available education programs, and clinical treatments. Regarding expanding the scope of KF-STRIDE, participants recommended including the following foci: general interview expectations, the use and interpretation of non-verbal communication during the interview, hygiene and appearance, other strategies to respond to common interview questions, and work-related skills. It’s noteworthy that although participants appreciated the strengths focus, they suggested that KF-STRIDE should also target areas of improvement (e.g., to work on improving deficits). The young adult on the spectrum expressed that only focusing on the positive would be ignoring potentially important areas that need addressing, and that he would appreciate being guided on what is being done wrong, in addition to what is done correctly. Other suggestions for KF-STRIDE included adding more visuals and reducing text to support those with co-occurring reading difficulties, increasing interaction opportunities between participants and the KF-STRIDE interventionist, preparing youth on the spectrum to work with an interviewer or interventionist, integrating the separate instructional materials into a streamlined curriculum, and providing guidance to support completion of assignments.

### 3.5. Modified KF-STRIDE

**Figure 2:**
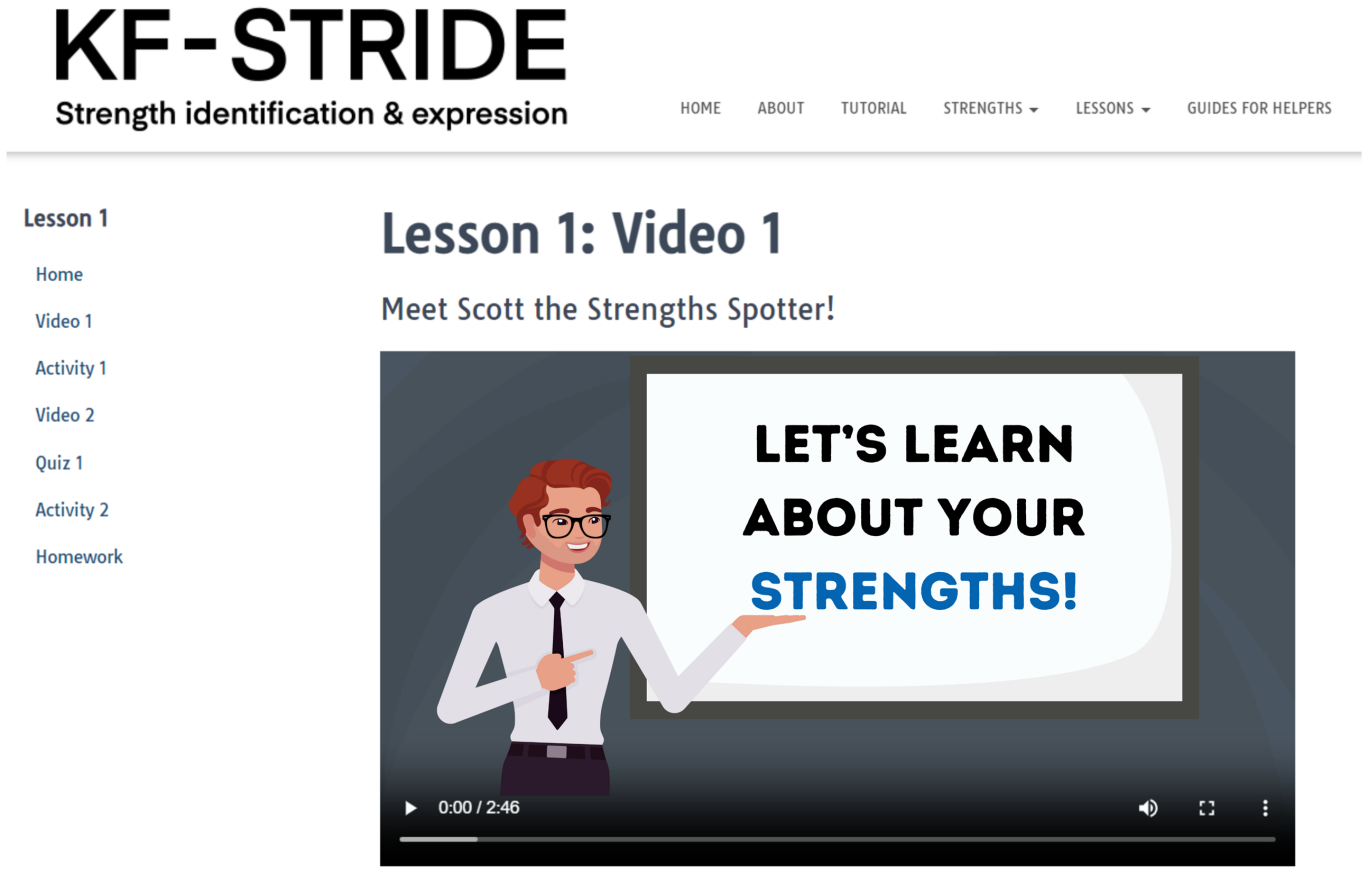
KF-STRIDE website featuring animated character to deliver content to user.

As a result of the stakeholder feedback, KF-STRIDE has been significantly modified. First, the original KF-STRIDE delivered content on powerpoint slides *via* an intervention. We have adapted KF-STRIDE to deliver its content *via* a multi-media website. Specifically, the KF-STRIDE lessons are now selected from a drop-down menu on the website, which includes a combination of web-based activities (e.g., answering self-reflective questions and performing practice interviews) and didactic content delivered through videos with an animated character, *Scott the Strength Spotter* (See Figure 2). *Scott the Strength Spotter* replaces the interventionist, as he delivers the content. The choice to have content delivered by Scott was made for several reasons. First, the use of *Scott the Strength Spotter* reduces the interventionist’s role, which increases the accessibility and scalability of KF-STRIDE, as it does not require intense training for an interventionist. This approach specifically addresses one of the feasibility barriers described in the interventionist feedback. Second, the use of the animated character ensures the standardized delivery of the content, thereby reducing potential bias by interventionists and minimizing implementation variability across settings.

Although *Scott the Strength Spotter* delivers the content, there is still a role for a supportive coach who is now called a “KF-STRIDE Guide.” In the newly modified KF-STRIDE, the role of the Guide is *not to deliver the content*. Rather, the Guide holds the trainee accountable by being present at all sessions and providing standardized feedback to the trainee (Guides are trained to fidelity *via* a manual). The presence of the Guide is based on a social cognitive theory principle of supportive accountability (41), based on research that indicates that adherence to behavioral programs are maximized by the presence of a coach (41, 42). In current evaluations, the Guide is a research assistant; however, training manuals have been developed so that teachers, parents and job coaches and others can become the KF-STRIDE Guide.

While KF-STRIDE is still based on the *Aware-Explore-Apply* positive psychology intervention model to improve identification of strengths, we have made several significant adaptations in response to stakeholder recommendations. For instance, KF-STRIDE now integrates a theory-of-mind based strategy. Theory-of-mind refers to understanding the beliefs, thoughts and expectations of others, which may differ from one’s own. Thus, participants will be first taught what the interviewer is expecting to hear (a description of their strengths), which may differ from what the participant wants to share (irrelevant, unexpected information) (43–46). Second, participants will repeatedly practice job interview role-plays in which they will be taught socially appropriate ways to relate information about their strengths (e.g., they are taught to relate their personal strengths to the job for which they are applying). In several studies, a similar theory-of-mind directed method has been successful in improving self-presentation in autistic individuals, increasing the number of “positive” self-referring statements compared to a condition in which no strategy was given (44–46). Our purpose in introducing this strategy is to reduce the amount of irrelevant information shared on a job interview, such as hobbies or facts about the interviewee that the employer is not expecting.

In addition, we modified KF-STRIDE to include new content focused on job interview skills beyond strength knowledge and expression. Specifically, participants are now taught about hygiene (e.g., showering the day of the interview), general interview etiquette (e.g., showing up on time), talking about their limitations in an appropriate way, researching the company ahead of time, asking appropriate questions, and writing a thank you note. This new content is introduced *via* the videos of *Scott the Strength Spotter* and include additional activities such as answering self-reflective questions and completing quizzes to reinforce what the participants have learned.

Another modification that we made was to increase the number of practice interviews available for trainees. Now, trainees first practice answering interview questions posed by an animated character. Then in further simulated interviews, participants will respond to pre-recorded videos of interviewers asking them strength-related questions (e.g., *“What strengths do you have to offer to this company*?”) using interview questions were derived from methods published by Smith et al. (38). To add realism, the interviews appear as though the trainees are participating in a web-based interview and interviewers are representative of varying races, genders and age groups. Throughout these practice interviews, the KF-STRIDE Guide gives feedback after each answer using concrete, scripted explanations as to why some of the responses were appropriate, off-topic, or irrelevant. See Table 4 for an example of feedback. In addition, in one lesson the participants film themselves performing a job interview, and then watch themselves and provide feedback to themselves with guidance from the KF-STRIDE Guide.

**Table 4:** Sample feedback provided during KF-STRIDE practice interviews.

Practice question: *Can you tell me about your strengths?*	
Possible response type	Feedback
Strength is mentioned and how it relates to job. Strength is mentioned with no relevance to job. Strength is not mentioned. Response is off-topic and not related to the question. Strength is not mentioned. Response portrays applicant as negative. Student does not answer	*“Great answer! Let us try the next question.”* *“Good job at mentioning your strength. Can you give an example of how this would be helpful for employment at this company?”* *“You mentioned thing(s) that were off topic. Let us try again! Remember, the interviewer really wants to hear about your strengths, try talking about one of your top three strengths.”* *“You talked about something that was negative. The employer does not expect that you will talk negatively about yourself or others. Let us try again but this time try to mention your strengths and stay positive.”* *“Well, let us try to think about the strengths we have discussed. Would you like to review them again?”*

The possible response types are provided in the left column, and their corresponding feedback is presented in the right column.

## 4. Discussion and conclusion

In the current study, we presented KF-STRIDE, a strengths-focused job interview training based on a positive psychology intervention framework. We examined both quantitative and qualitative data to identify whether KF-STRIDE was acceptable, usable, and feasible. Qualitative data collected from two autistic individuals, educators, therapists, a parent, an intervention scientist, and interventionists provided valuable feedback which guided modifications to KF-STRIDE to ensure that it better suits the needs of the autism community.

The quantitative and qualitative data collected from young adults on the spectrum who participated in the training indicated that the intervention is well-accepted. KF-STRIDE was viewed as a valuable tool for improving job interview skills and confidence. More importantly, stakeholder feedback indicated KF-STRIDE may have more benefits beyond job interview skills; it could inform individuals about strengths they may not know about themselves which could potentially improve self-esteem and social skills. This finding is in line with recent trends in the disability field to focus on the cultivation of strengths to improve well-being (35, 47). Specifically in the autism community, strength-based practices are increasing in popularity as they are thought to be effective as well as well-accepted (48–50).

Stakeholder feedback was used to guide important modifications to KF-STRIDE. Notably, KF-STRIDE was translated from an interventionist-led program implemented *via* power-point to a web-based intervention where content is delivered by an animated character, an approach which increases its potential for accessibility and scalability. Scott the Strength Spotter’s (the animated character) role is to deliver the content of the animation, thereby reducing the burden and resources associated with a clinician or job-coach delivering the intervention. By including a human in the role of a support coach (KF-STRIDE Guide), we are able to continue to ensure participant engagement and adherence to the protocol.

Another major change to the KF-STRIDE program is the introduction of a theory-of-mind based strategy. This change was made to address concerns that while participants may provide personal information on a job interview, that information may be inconsistent with what the employer is expecting to hear. For example, while speaking about one’s favorite video game may be appropriate in an informal conversation, it may not be appropriate or strategic in a job interview. Thus, the theory-of-mind based strategy was to first inform the users what is expected on a job interview (to speak positively about one’s strengths), and then to allow the users to practice self-presentation in a structured way while receiving feedback from the program. Related to this, additional opportunities to practice answering job questions is now incorporated into the newly modified KF-STRIDE.

Additional skills beyond learning about strength expression are now taught during the modified KF-STRIDE program, such as job interview etiquette. While answering job interview questions appropriately and strategically is the critical goal of KF-STRIDE, teaching users to write appropriate thank-you notes, practice personal hygiene and arrive punctually were important skills suggested by stakeholders. These skills are often taught in employment based training programs for autism.

Limitations of this study include a small sample size which limits our ability to generalize the findings to the broader autism population. The youth that were included in the study may also have not been representative of job-seeking individuals on the spectrum given their lack of employment history and job search behavior. Therefore, evaluating the acceptability and efficacy of KF-STRIDE in a larger sample is a necessary next step in our evaluation.

### 4.1. Conclusions and future directions

Community engaged methods to guide intervention development are rapidly becoming an essential part of the behavioral research process. We developed KF-STRIDE using a positive psychology framework in order to increase strength awareness in young adults on the spectrum within the context of job interviews. This evaluation generated critical feedback from the autism community and others that helped guide our modifications to increase the acceptability, usability, and feasibility of KF-STRIDE for the autism community. Our future directions include fully evaluating the modified KF-STRIDE for acceptability, usability, implementation feasibility, and effectiveness. As autism services research continues to seek strength-based approaches, there is a critical need for evidence-based interventions to fill this gap in our approach to youth on the spectrum.

## Data availability statement

The raw data supporting the conclusions of this article will be made available by the authors, without undue reservation.

## Ethics statement

The studies involving humans were approved by Kessler Foundation Institutional Review Board. The studies were conducted in accordance with the local legislation and institutional requirements. Written informed consent for participation in this study was provided by the participants’ legal guardians/next of kin.

## Author contributions

HG was responsible for conceptualization, data curation, formal analysis, investigation, methodology, project administration, resources, supervision, visualization, writing—original draft, and writing—review and editing. MH was responsible for data curation, formal analysis, methodology, project administration, visualization, writing—original draft, and writing—review and editing. Y-LC was responsible for formal analysis, visualization, writing—original draft, and writing—review and editing. HE was responsible for formal analysis, visualization, and writing—review and editing. RM and MS were responsible for conceptualization and writing—review and editing. All authors contributed to the article and approved the submitted version.

## Funding

This study was supported by National Institute of Mental Health grant to HG (K18 MH122847). This work was also supported by the Reitman Foundation, the New Jersey Governor’s Council for Medical Research and Treatment of Autism under grant CAUT19APL027.

## Conflict of interest

The authors declare that the research was conducted in the absence of any commercial or financial relationships that could be construed as a potential conflict of interest.

## Publisher’s note

All claims expressed in this article are solely those of the authors and do not necessarily represent those of their affiliated organizations, or those of the publisher, the editors and the reviewers. Any product that may be evaluated in this article, or claim that may be made by its manufacturer, is not guaranteed or endorsed by the publisher.

## Footnotes

^1^ https://www.researchmatch.org/
